# Optical clearing with tartrazine enables deep transscleral imaging with optical coherence tomography

**DOI:** 10.1117/1.JBO.29.12.120501

**Published:** 2024-12-09

**Authors:** Amit Narawane, Robert Trout, Christian Viehland, Anthony N. Kuo, Lejla Vajzovic, Al-Hafeez Dhalla, Cynthia A. Toth

**Affiliations:** a Duke University, Department of Biomedical Engineering, Durham, North Carolina, United States; b Duke University Medical Center, Department of Ophthalmology, Durham, North Carolina, United States

**Keywords:** tartrazine, optical coherence tomography, optical clearing, suprachoroidal injections

## Abstract

**Significance:**

Imaging deep structures with optical coherence tomography (OCT) is difficult in highly scattering biological tissue, such as the sclera. There is a need to visualize the suprachoroidal space and choroid through the sclera to study suprachoroidal drug delivery.

**Aim:**

We aim to develop optical methods to image through the highly scattering sclera with a custom-built OCT system to visualize the suprachoroidal space and drug delivery within.

**Approach:**

We developed a custom handheld OCT scanner to image the anterior segment and suprachoroidal space in *ex vivo* eye models. Tartrazine (Yellow 5) solution, which has been shown to optically clear biological tissue in the visible regime, was tested as a clearing agent to optimize near infrared OCT imaging through the sclera.

**Results:**

Tartrazine dramatically increased OCT signal return from the deeper sclera and choroid and thus enabled visualization of the suprachoroidal drug delivery after transscleral injection.

**Conclusions:**

We demonstrated successful optical clearing of the thick, porcine sclera with a compact handheld OCT system to image the suprachoroidal space. We believe there is broader potential to use optical clearing with handheld OCT for a variety of previously inaccessible, highly scattering tissue samples.

## 1 Introduction

Retinal diseases such as age-related macular degeneration, diabetic retinopathy, and uveitic macular edema are leading causes of visual impairment.^1^^–^^3^ Intravitreal injections of anti-vascular endothelial growth factor (VEGF) agents and/or steroids have been effective in controlling vascular leakage and often restoring visual acuity but require repeated injections. Novel treatments such as intravitreal reservoirs or subretinal injections of gene therapies can be effective over a longer period but may cause glaucoma and cataract (in the case of steroids) or incur the cost and complexity of delivery in a microsurgical setting (in the case of subretinal gene therapy).^4^ This has led to the development of ocular therapeutics that can be delivered by transscleral injection into the suprachoroidal space in an office-based procedure.^5^ Triamcinolone suspension via suprachoroidal injection was approved in 2021 by the U.S. Food and Drug Administration for the treatment of uveitic macular edema, and clinical trials are underway using this delivery route for other posterior segment diseases. A limitation of this procedure is that clinicians are unable to obtain real-time feedback on the location and spread of the delivered product; rather, heat map imaging is used to estimate this.^5^

Optical coherence tomography (OCT) has widespread use in ophthalmology and allows for micron-scale depth resolution of the anterior eye.^6^^,^^7^ We have previously demonstrated visualization of drug delivery during subretinal injections with intraoperative OCT systems.^8^ We hypothesize that OCT also has the potential to visualize drug delivery to the suprachoroidal space. To do this, transscleral imaging is required, which necessitates ballistic photon travel through the sclera, a tissue composed of dense, randomly oriented collagen.^9^ This medium is highly scattering at near-infrared (NIR) wavelengths utilized by OCT systems,^10^ limiting the imaging depth to 1 to 2 mm, which is insufficient to visualize the suprachoroidal space.^11^^,^^12^

To improve OCT signal return from greater depths in scattering tissue, several hardware-based approaches have been applied, including offset illumination and collection pathways to recover multiply scattered light^13^ and ultrasound-based mechanical clearing of tissue.^14^ However, these methods require significant augmentation of the imaging system. As an alternative, chemical approaches involving biocompatible tissue clearing agents have been investigated for potential in OCT imaging, including various sugars, glycerol, propylene glycol, and dimethylsulfoxide.^11^^,^^15^^–^^17^ Their mechanism consists of introducing the clearing agent into the interstitial fluid of the tissue via topical exposure. The homogenization of refractive index in the interstitial fluid results in optical clearing and reduced scattering. However, the efficiency of refractive index change for these agents is poor, requiring high concentrations within the tissue to achieve substantial clearing. Recently, tartrazine (Yellow 5) has been shown to have potential as a more efficient biocompatible optical clearing agent for biomedical tissue imaging with brightfield, fluorescence, laser speckle contrast, and second harmonic generation microscopy.^18^ Motivated by these findings in visible light, we endeavored to investigate whether tartrazine could be used to improve near-infrared OCT depth penetration in highly scattering tissue such as the sclera; in this case, to facilitate improved visualization of suprachoroidal injection dynamics.

In this letter, we describe transscleral imaging with and without tartrazine using a custom handheld telecentric OCT probe designed for high-resolution imaging of the anterior eye. We first evaluated our OCT probe for imaging the anterior segment by correlating imaging results from *ex vivo* human eyes to known anatomical structures seen in histology. We then demonstrated in *ex vivo* porcine eyes that tartrazine optically clears scleral tissue for our infrared imaging bandwidth to improve the depth penetration of OCT. To our knowledge, this work presents the first use of tartrazine as an optical clearing agent for OCT. We believe these methods will enable OCT studies in thick, scattering samples that were previously optically limited or inaccessible.

## 2 Materials and Methods

### 2.1 Handheld Anterior Segment OCT System

**Fig. 1:**
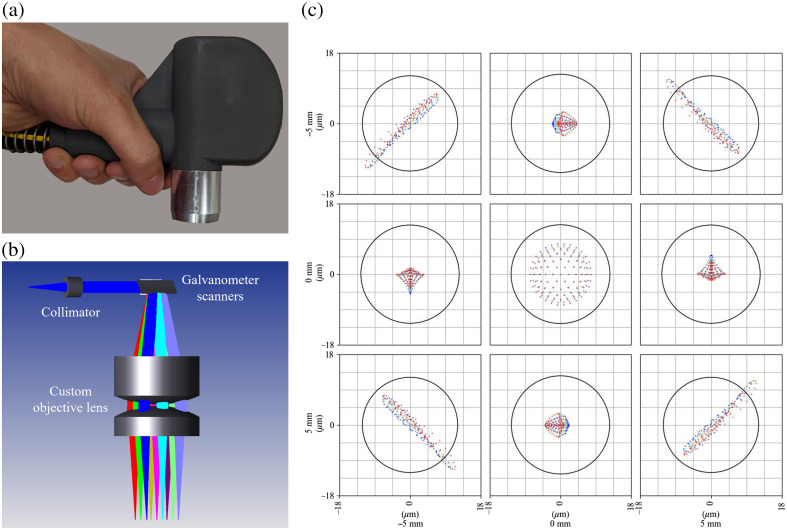
Schematic of custom handheld OCT system. (a) Photo of the real device. (b) Zemax OpticStudio model of the design with nine representative scan configurations. (c) Spot diagrams from each scan configuration at the image plane for a 10×10  mm field of view (12.7  μm Airy radius).

Traditional tabletop OCT systems are difficult to use for real-time imaging of procedures, such as suprachoroidal injections, in the research lab or the clinic. We developed a purpose-built handheld OCT system, including a telecentric sample arm with custom-designed optics and opto-mechanics (Fig. 1). The probe is highly compact and functions in both handheld and table-mounted modes. Our design provides a 25-mm working distance, lateral resolution of 12.7  μm (Airy radius), and lateral field of view >18  mm. The OCT engine employs a swept-source laser operating at 1040 nm with a 104-nm bandwidth and a 200-kHz sweep rate (Excelitas, Axsun Technologies Inc., Billerica, Massachusetts, United States), delivering an axial resolution of 5.92  μm (in air) and average sample arm power of 1.23 mW. Our scan protocol imaged a field of view (FOV) of 13×13  mm, with 1000 A-scans/B-scan and 128 B-scans/volume, and 8× repeated B-scans for multi-frame averaging. This OCT system is well-suited for use in imaging and evaluating suprachoroidal injections both *ex vivo* and in the clinic.

### 2.2 Imaging and Optical Clearing in *Ex Vivo* Eye Models

To validate our custom OCT system for anterior segment imaging and identification of anterior segment structures, we performed a preliminary experiment on post-mortem human donor eyes. The eyes were marked with sutures that acted as fiducial markers among images. The eyes were imaged across the limbus in the superotemporal quadrant with our OCT system. We then fixed the eyes in formalin for 24 h followed by paraffin embedding, sectioning, and hematoxylin and eosin (H&E) staining. The sutures were used to align B-scans with their corresponding histological sections.

**Fig. 2:**
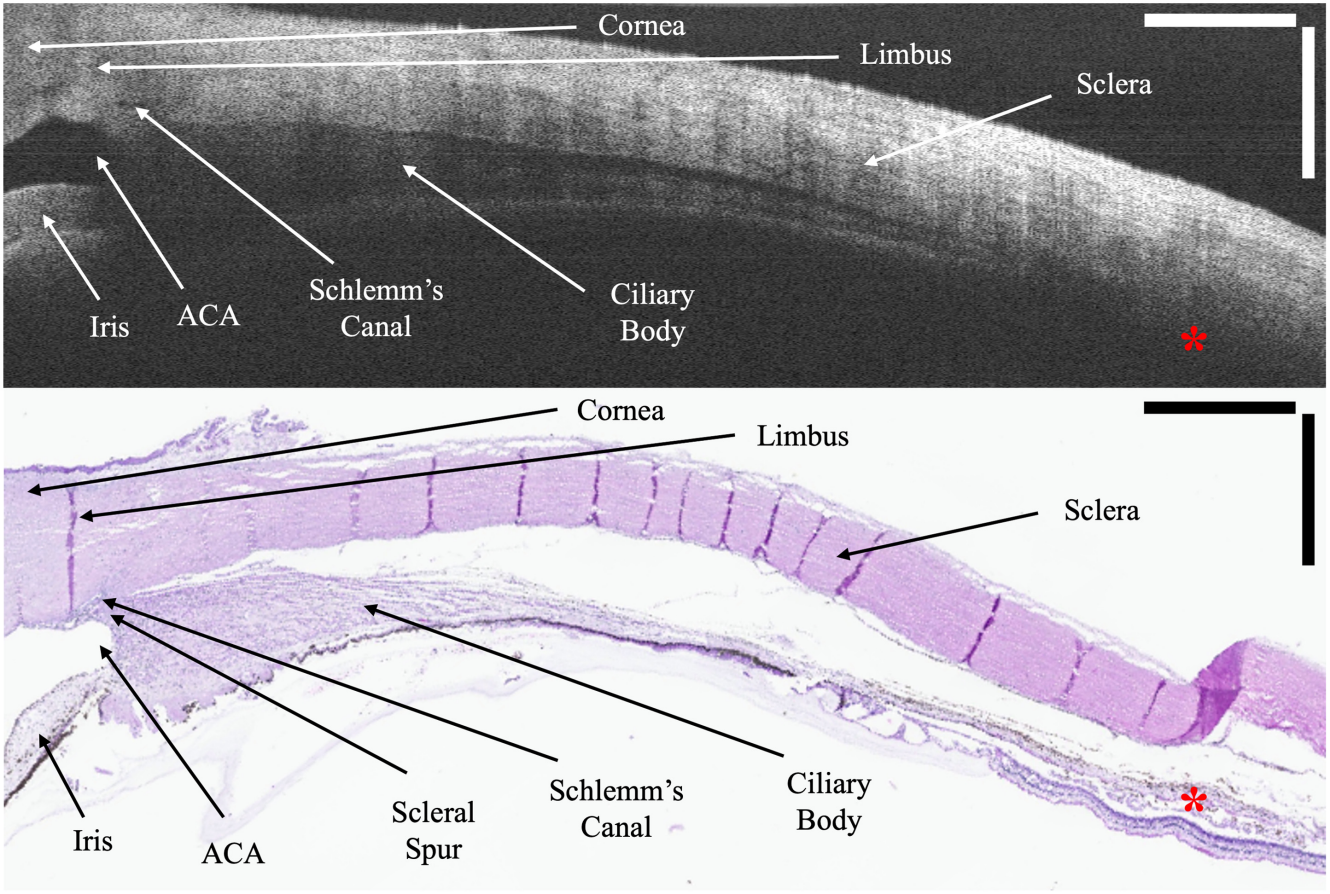
Correlation of anterior segment features between an OCT B-scan (8× averaged) and the corresponding H&E section. The conjunctiva and Tenon’s complex were removed prior to imaging. Tissue separation is present in OCT due to the multi-day transit of donor eyes and more so in the H&E section due to processing. Red asterisks highlight the lack of choroidal/suprachoroidal visualization through the sclera on OCT. ACA, anterior chamber angle. Scale bar is 1 mm in tissue.

To test optical clearing in OCT, a pair of *ex vivo* porcine eyes, derived from the same animal to ensure comparability, were used in this experiment. Porcine eyes are preferred for iterative studies as they are readily available fresh from an abattoir. There is often a delay post-mortem in obtaining donor human eyes, during which choroidal and retinal separation is common (as seen in Fig. 2). This makes it difficult to determine whether the suprachoroidal space is visible due to injection or tissue separation. Porcine eyes can be acquired immediately after death, which makes them a superior model for studying real-time suprachoroidal injections. Although the porcine sclera from small animals has been reported to be comparable in thickness to that of the human,^19^ the sclera of our specimens has been thicker (consistent with the larger source pigs from 200 to 360 kg), ranging from 1-mm-thick at 8 mm posterior to the limbus in the superotemporal quadrant to 2- to 3-mm-thick across most other regions.

One globe was immersed in a 0.4-M tartrazine solution. At 0.4 M, 1.6 Osm, this tartrazine solution is hyperosmotic to tissue (1.6  Osm>0.3  Osm).^20^ Hyperosmotic solutions have a dehydrating effect on tissue that can confound results with increased imaging depth. To distinguish between dehydration and optical clearing, the second paired globe was immersed in a saline solution of equal osmolarity to the tartrazine solution (0.8 M NaCl, 1.6 Osm) as a control. Each eye was immersed for a total of 120 min, with imaging time points at 0, 10, 20, 30, 60, and 120 min. At each time point, the immersion solution was temporarily removed for ∼1  min to expose the eye for imaging. Both eyes were imaged in the temporal region.

To quantify the change in depth penetration with tartrazine, we reported changes in relative signal level across scleral depth. The central 10 A-scans of each image at each time point were averaged, and the smoothed result was cropped at the scleral surface. To assess the visualization of suprachoroidal drug delivery with optical clearing, we performed suprachoroidal injections on the porcine eyes with and without exposure to tartrazine and acquired OCT volumes before and after each injection. Injections were 0.1 mL of saline or steroid solution and were performed using the SCS Microinjector (Clearside Biomedical, Alpharetta, Georgia, United States).

## 3 Results

Figure 2 shows the correlations between an OCT B-scan and its corresponding H&E section in *ex vivo* human donor eyes using known anterior segment anatomical features.^21^ Although some anterior structures can be identified on OCT, it is difficult to clearly resolve deeper structures such as the ciliary body and scleral spur. As the sclera becomes thicker farther from the limbus, OCT is completely unable to visualize the choroid or the boundaries of the suprachoroidal space (red asterisks).

**Fig. 3:**
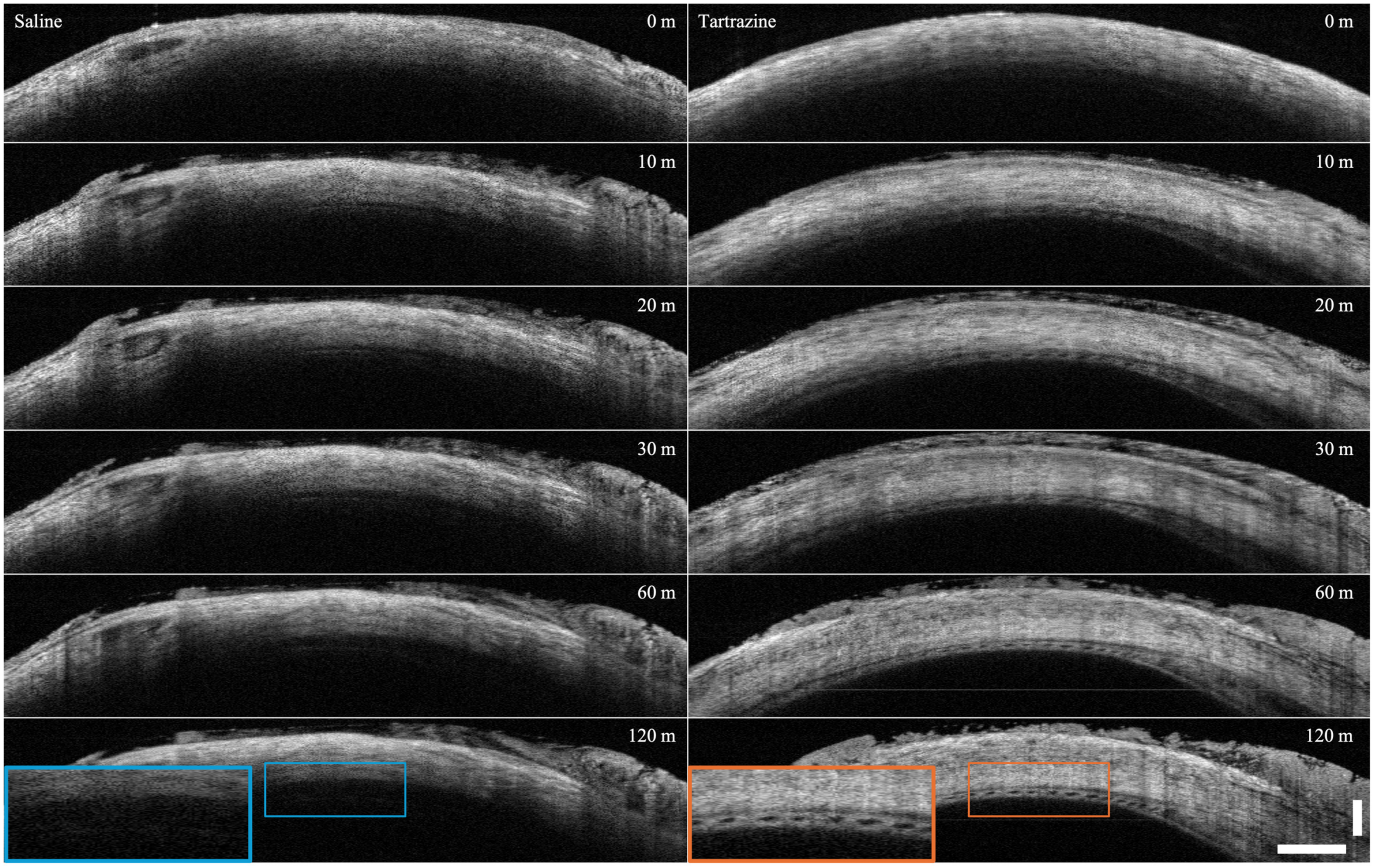
OCT B-scans (8× averaged) from a pair of *ex vivo* porcine eyes from the same animal. One eye from the pair (left column) is immersed in saline and the other (right column) in tartrazine. Each eye was imaged at the same location at 0, 10, 20, 30, 60, and 120 min. Blue and orange insets show expected depth-related loss of signal in the control saline eye (blue) and revealed choroid boundaries and large choroidal vessels with tartrazine treatment (orange). Scale bar is 1 mm in tissue.

Figure 3 shows the representative B-scans from the *ex vivo* porcine eyes immersed in saline and tartrazine, captured and processed with the same protocol described above at each time point but with an increased FOV of 18 × 18 mm. Although the eye in saline showed minimal change from start to finish, even with 8× multi-frame averaging, the eye in tartrazine showed dramatically increased depth penetration of OCT light through the sclera over time. At the 2-h mark, we achieved near-full visualization of the choroid through the sclera, with individual choroidal vessels visible that were completely unseen in the control eye.

**Fig. 4:**
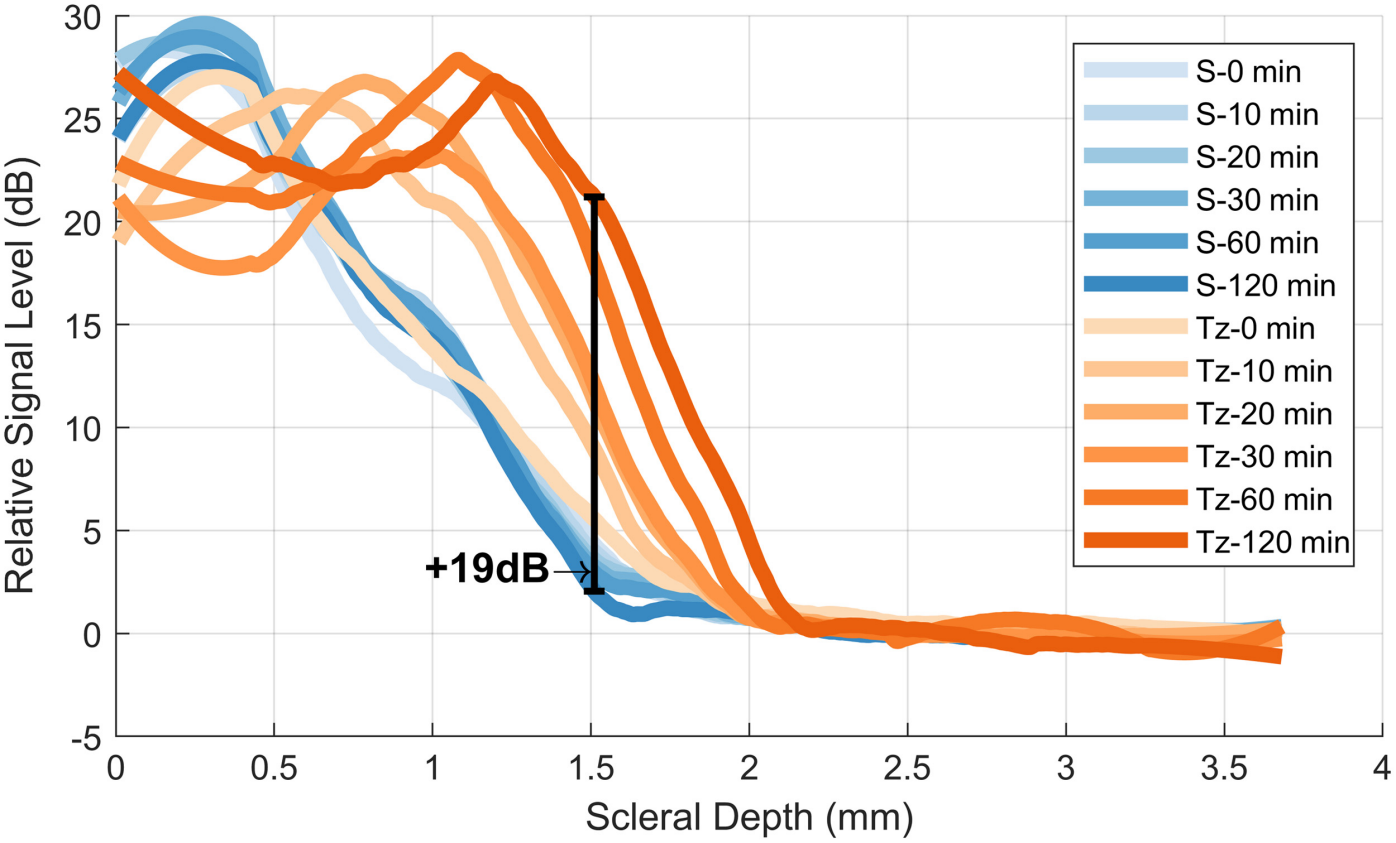
Relative signal level across depth for central 10 A-scans of each image in Fig. 3. Maximal signal increase between S-120 min and Tz-120 min is annotated. S, saline; Tz, tartrazine.

Figure 4 shows the comparison of signal return across depth from the center of the images shown in Fig. 3. Here, we see increased signal return at greater depths in the tartrazine exposed eye than we do in the control eye. We note that the dip in signal in the shallow parts of the sclera is expected; as the tissue is surrounded by a material with a high refractive index, less light is reflected by the shallower parts of the sclera and instead penetrates further into the tissue before being back-scattered at a deeper layer. The black bracket highlights the maximum observed increase in relative signal of 19 dB (86-fold increase in return power) located at a depth of approximately 1.5 mm.

**Fig. 5:**
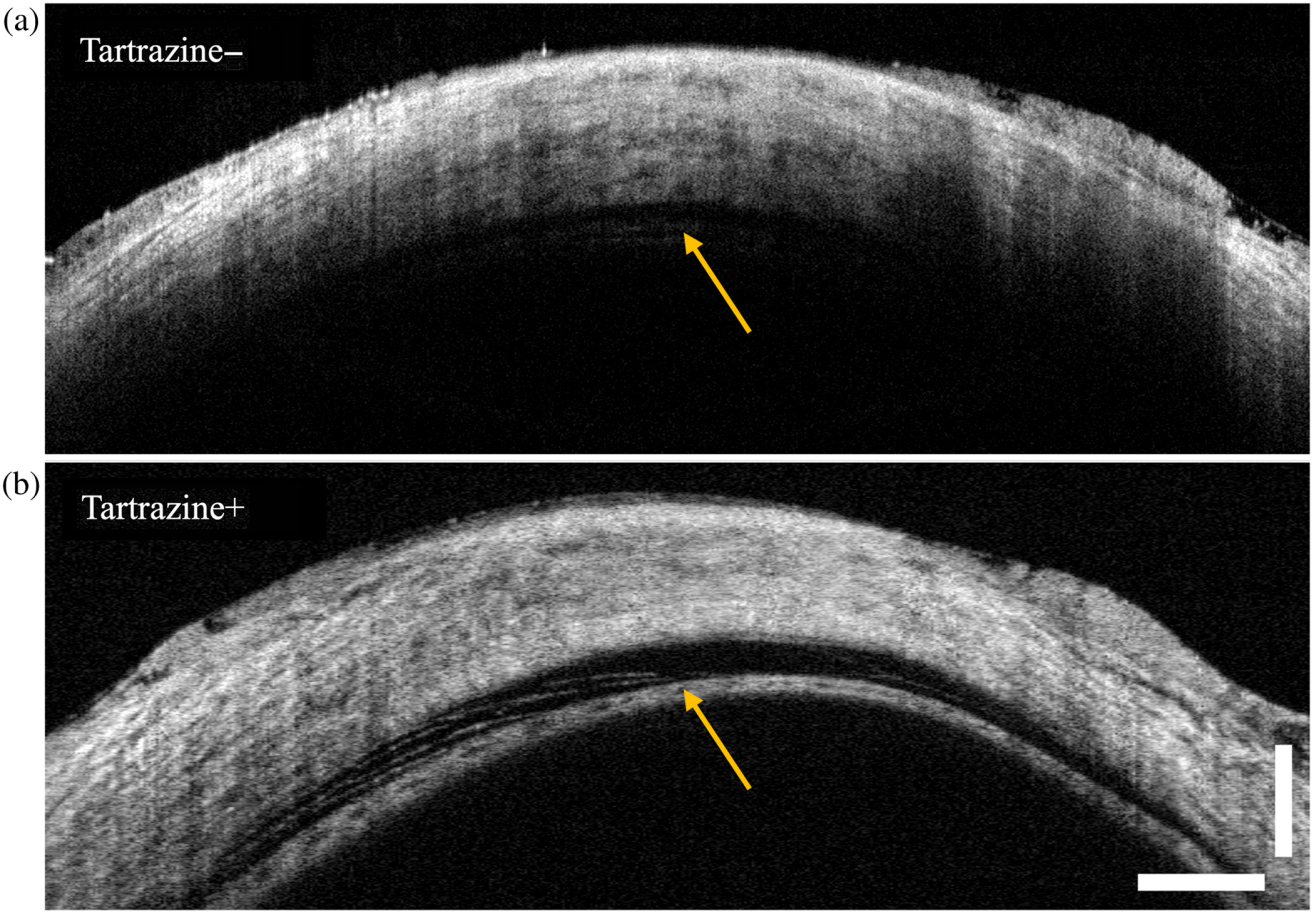
Representative OCT B-scans (8× averaged) captured after suprachoroidal injection in *ex vivo* porcine eyes (a) without tartrazine and (b) with 2-h tartrazine immersion. Yellow arrows indicate the location of the choroidal boundary with the suprachoroidal space above it after injection, in contrast to the absence of the space prior to injection (Fig. 3). The signal in the tartrazine eye reveals the strands of reflective tissue bridging the suprachoroidal space (left of the arrow) and the broad extent of choroidal separation. Scale bar is 1 mm in tissue.

OCT B-scans after suprachoroidal injection are shown in Fig. 5. Although the lower boundary of the suprachoroidal space is difficult to visualize in the first eye, the same boundary is clearly seen once the sclera has been optically cleared with tartrazine.

## 4 Discussion and Conclusion

In this work, we have achieved the first demonstration of optical clearing in OCT using highly absorbing molecules. Our results show that the methods described by Ou et al.^18^ are extendable to OCT imaging and can likely be applied to any reflectance-based imaging modality. We have also demonstrated that our custom handheld anterior segment OCT design is capable of scleral imaging and visualization of the suprachoroidal space and of the choroid and choroidal vasculature with optical clearing agents.

Although tartrazine had a large effect on optical clearing in transscleral OCT, it is possible that further improvement could be seen with the application of another food grade dye with a more red-shifted absorption spectrum, such as Allura-Red (Red 40), which may result in greater optical clearing effect in the NIR region of OCT imaging.

Here, we investigated *ex vivo* tissues, and the logical next step would be to conduct *in vivo* studies on animal models to validate the method in a living system. There may be challenges with living vasculature removing the clearing agent more rapidly than in *ex vivo* samples, however with the potential benefit of improved visualization of *in vivo* injection dynamics. We anticipate this work will enable further study of transscleral imaging of the suprachoroidal space, with the eventual goal of improving the visibility of suprachoroidal injection therapies in humans.

## Data Availability

Data underlying these results are available upon reasonable request.
