# Publisher’s Note: Optical clearing with tartrazine enables deep transscleral imaging with optical coherence tomography

**DOI:** 10.1117/1.JBO.29.12.129801

**Published:** 2024-12-18

**Authors:** Amit Narawane, Robert Trout, Christian Viehland, Anthony N. Kuo, Lejla Vajzovic, Al-Hafeez Dhalla, Cynthia A. Toth

**Affiliations:** a Duke University, Department of Biomedical Engineering, Durham, North Carolina, United States; b Duke University Medical Center, Department of Ophthalmology, Durham, North Carolina, United States

## Abstract

The note clarifies a correction to the citation information.

The article was erroneously published in Volume 29 Issue S2 of *Journal of Biomedical Optics* (JBO) on 9 December 2024. It was subsequently re-published a few days later (12 December 2024) in Issue 12 of the same volume and the citation identifier was corrected from S20501 to 120501. Correct citation for this article includes the updated DOI: 10.1117/1.JBO.29.12.120501.

